# Pregabalin prescription for terminally ill cancer patients receiving specialist palliative care in an acute hospital

**DOI:** 10.1186/s40780-016-0063-6

**Published:** 2016-11-05

**Authors:** Ryo Yajima, Kazuaki Matsumoto, Yuya Ise, Norihito Suzuki, Yuta Yokoyama, Junko Kizu, Shiro Katayama

**Affiliations:** 1 Section of Pharmaceutical Services, Nippon Medical School Hospital, 1-1-5 Sendagi, Bunkyo-ku, Tokyo, 113-8603 Japan; 2 Division of Practical Pharmacy, Keio University Faculty of Pharmacy, 1-5-30 Shibakoen, Minato-ku, Tokyo, 105-8512 Japan; 3 Department of Anesthesiology, Nippon Medical School, 1-1-5 Sendagi, Bunkyo-ku, Tokyo, 113-8603 Japan; 4 Department of Palliative Care, Nippon Medical School Hospital, 1-1-5 Sendagi, Bunkyo-ku, Tokyo, 113-8603 Japan

**Keywords:** Pregabalin, Adjuvant analgesic, Cancer pain, Terminally ill, Palliative care team

## Abstract

**Background:**

Pregabalin is recommended as an adjuvant analgesic for neuropathic cancer-related pain, and may be taken at all steps of the World Health Organization analgesic ladder. However, unlike opioids, pregabalin treatments are limited to an oral administration route. If patients have oral feeding difficulties, it is not possible to administer any drug as an adjuvant analgesic for neuropathic cancer-related pain. Therefore, the aim of the present study was to clarify the problems of pain control after pregabalin discontinuation in terminally ill cancer patients.

**Methods:**

Our subjects comprised cancer patients who died during their hospital stay and were referred between April 2013 and October 2015 to the palliative care team of the 899-bed Cancer Hospital at the Nippon Medical School Hospital in Japan. The medical records of each patient were retrospectively reviewed, and patient characteristics were recorded.

**Results:**

We obtained data on 183 patients during the study period. Thirty-eight (20.8 %) patients were treated with pregabalin. Thirty-three (86.8 %) out of 38 patients were prescribed pregabalin for neuropathic cancer-related pain. The incidence of bony metastases was significantly higher in patients administered pregabalin than in those not taking the drug (non-pregabalin group 32.4 % vs pregabalin group 57.9 %). Pregabalin was ultimately discontinued in all patients, with the main reason being oral feeding difficulties (81.6 %). After the discontinuation of pregabalin, the amount of opioid drugs administered was increased in 56.5 % of patients with oral feeding difficulties.

**Conclusion:**

Our results demonstrated that the amount of opioid drugs administered was increased in more than 50 % of patients following the discontinuation of pregabalin, and was repeatedly increased for some patients. A new administration route is required for cancer patients unable to take oral medication.

**Trial registration:**

UMIN000022507. May 28, 2016 retrospectively registered.

## Background

Pain occurs in approximately 30 % of all cancer patients, and in 60–70 % and 75 % of advanced and terminally ill cancer patients, respectively. Persistent pain is the most common type of pain reported in cancer patients; 50 % of patients have moderate or advanced pain, while 30 % have advanced or unbearable pain [1, 2]. Since terminally ill cancer patients have emotional distress and physical pain, palliative care incorporates the administration of a number of different drugs including opioids, antipyretic analgesics, and adjuvant analgesics.

Cancer cells in terminally ill cancer patients spread to various sites. When metastasized cancer cells compress nerve tissue, characteristic pain, such as electric shock-like pain with tingling, develops. Pregabalin is recommended as the first-line therapy in the guidelines for neuropathic pain, is widely used as an adjuvant analgesic for patients with neuropathic pain, and may be taken at all steps of the World Health Organization analgesic ladder [3, 4].

However, unlike opioid drugs and antipyretic analgesics, pregabalin treatments are limited in Japan to an oral administration route. If terminally ill patients develop oral feeding difficulties, it is not possible to administer any drug as an adjuvant analgesic for neuropathic cancer-related pain. Therefore, pain control has not yet been established for terminally ill cancer patients with oral feeding difficulties.

The aim of the present study was to clarify the problems of pain control after pregabalin discontinuation. Therefore, we investigated the current status of pregabalin treatments in terminally ill cancer patients.

## Methods

### Data sources and procedures

The method used in the present study was a chart review. Subjects comprised patients with cancer who were referred between April 1, 2013 and October 31, 2015 to the palliative care team of the 899-bed Cancer Hospital at the Nippon Medical School Hospital in Japan. Inclusion criteria were: a diagnosis of incurable advanced cancer and patients who died during their hospital stay at Nippon Medical School Hospital.

### Demographic and patient clinical data

Information including age, sex, the date of admission, date of death, primary cancer site, bone metastases, requested reasons for palliative care, performance status (PS) at the time of the palliative care intervention, and a prescription for pregabalin was extracted from the electronic medical records of each patient.

### The current status of pregabalin treatments

Information including the prescriber, reasons for the prescription, initial dose, maintenance dose, administration period, weight, creatinine clearance (Ccr) calculated from the Cockcroft-Gault formula, if pregabalin had been discontinued, the reason for its discontinuation, and the dose of opioid drugs before and after the discontinuation of pregabalin was extracted from the electronic medical records of patients prescribed pregabalin.

We investigated the dose of opioids administered before and after the discontinuation of pregabalin. The doses of opioid drugs were converted to morphine-equivalent dose. The half-life of pregabalin according to renal function is 5–48 h, as stated on the package insert. Therefore, based on its excretion from the body, patients who survived 4 days or more after the discontinuation of pregabalin were targeted. The initial and maintenance doses were compared to doses on the package insert. The initial dose of pregabalin (daily dose), as recommended on the package insert, in the case of Ccr (mL/min) ≥ 60 was 150 mg, in the case of 60 > Ccr ≥ 30 was 75 mg, in the case of 30 > Ccr ≥ 15 was 50 mg, and in the case of Ccr < 15 was 25 mg. The maintenance dose (daily dose) and highest dose (daily dose) in each case of Ccr ≥ 60 were 300 mg and 600 mg, respectively, in each case of 60 > Ccr ≥ 30 were 150 mg and 300 mg, respectively, in each case of 30 > Ccr ≥ 15 were 75 mg and 150 mg, respectively, and in each case of Ccr < 15 were 50 mg and 75 mg, respectively.

### Statistical analyses

The Mann–Whitney U-test and chi-squared test were used to examine differences between patients prescribed and those not prescribed pregabalin. All analyses were performed with the Statistical Package for the Social Sciences (version 20.0, MAKER, LOCATION). The significance level was set at *P* < 0.05.

### Ethical issues

This study was approved by the Ethical Review Board of the Nippon Medical School Hospital (#28-05-580). Patient information was coded by number, and could not be identified personally.

## Results

### Background

We obtained data on 183 patients during the study period. Thirty-eight (20.8 %) patients were being treated with pregabalin. Each patient was separated by the presence or absence of a prescription for pregabalin, and patient backgrounds are shown in Table 1. The incidence of bony metastases was significantly higher in patients administered pregabalin than in those not taking the drug (Table 1).

**Table 1:** Patient characteristics

	Pregabalin prescription		*P* value
	Yes	No	
Patients in each group (N)	38	145	
Age (years, SD)	65.7 (13.8)	66.7 (10.5)	0.914^a)^
Male (N, %)	19 (50.0)	96 (66.2)	0.089^b)^
Duration of the hospital stay (day, SD)	43.8 (49.4)	38.0 (39.0)	0.668 ^a)^
Primary sites (N)			
Stomach/colon, rectum	8	61	
Liver/bile duct/pancreas	2	40	
Kidney/ureter	11	14	
Lung	2	9	
Ovary/uterus	3	8	
Head and neck/	1	5	
Blood	3	3	
Breast	1	2	
Others	7	3	
Bone metastases (N, %)	22 (57.9)	47 (32.4)	0.014 ^b)^
Requested reasons for palliative care ^c)^ (N)			
Pain	35	128	
Difficulty breathing	1	13	
Malaise	2	6	
Nausea	0	2	
Others	0	4	
PS (SD)	3.3 (0.6)	3.3 (0.7)	0.829 ^a)^

*SD* Standard deviation

^a)^ Mann–Whitney U-test, ^b)^ the chi-squared test, ^c)^Duplicate Yes

### Prescription for pregabalin

Twenty-five out of 38 patients were prescribed pregabalin by the Department of Palliative Care. Thirty-three out of 38 patients had been prescribed pregabalin for pain and numbness due to cancer (Table 2).

**Table 2:** Pregabalin prescribers and reasons for its prescription

Diagnosis and treatment department	Number of patients (*N*)
Palliative care	25
Attending physicians	8
Other (orthopedics)	3
Unknown (Continuous use from another hospital)	2
Prescription reason	
Cancer-related pain and numbness	33
Numbness due to an anti-cancer drug treatment	1
Numbness due to radiation therapy	1
Numbness after shingles	1
Pain associated with fibromyalgia syndrome	1
Unknown	1

### Initial and maintenance doses of pregabalin

A comparison of the initial dose of pregabalin administered to patients and the initial dose on the package insert revealed that the percentage of patients started on a smaller dose than that on the package insert in the case of Ccr ≥ 60 was 61.1 %, in the case of 60 > Ccr ≥ 30 was 69.2 %, in the case of 30 > Ccr ≥ 15 was 66.7 %, in the case of Ccr < 15 was 0.0 %, and for all cases was 62.9 %. The maintenance dose was compared in the same manner. The percentage of patients started on a smaller dose than that on the package insert in the case of Ccr ≥ 60 was 91.7 %, in the case of 60 > Ccr ≥ 30 was 70.0 %, in the case of 30 > Ccr ≥ 15 was 0.0 %, in the case of Ccr < 15 was 100.0 %, and for all cases was 72.2 % (Table 3).

**Table 3:** The number of patients with initial dose and maintenance dose (mg/day) of pregabalin

	Ccr (mL/min)			
	Ccr ≥ 60	60 > Ccr ≥ 30	30 > Ccr ≥ 15	Ccr < 15
Initial dose ^a)^				
150 mg	7	2	0	0
75 mg	5	2	1	0
50 mg	2	2	0	0
25 mg	4	7	2	1
Maintenance dose ^b)^				
300 mg	1	3	0	0
250 mg	1	0	0	0
225 mg	2	0	0	0
150 mg	4	3	1	0
100 mg	0	1	0	0
75 mg	3	4	2	0
50 mg	0	1	0	0
25 mg	1	8	0	1

^a)^ The initial dose (mg/day) of pregabalin in the package insert, in the case of Ccr (mL/min) ≥ 60, was 150 mg, in the case of 60 > Ccr ≥ 30, was 75 mg, in the case of 30 > Ccr ≥ 15, was 50 mg, and in the case of Ccr < 15, was 25 mg

^b)^ The maintenance dose (mg/day) of pregabalin in the case of Ccr (mL/min) ≥ 60 was 300 mg, in the case of 60 > Ccr ≥ 30 was 150 mg, in the case of 30 > Ccr ≥ 15 was 75 mg, and in the case of Ccr < 15 was 50 mg

### Reasons for the discontinuation of pregabalin

Pregabalin was ultimately discontinued in all patients, with the main reason being oral feeding difficulties (81.6 %) (Table 4). Six patients discontinued pregabalin due to side effects.

**Table 4:** Reasons for discontinuing pregabalin

Reasons for discontinuing	Number of patients (N)
Oral feeding difficulty	31
Adverse effect (sleepiness)	2
Adverse effect (renal dysfunction)	2
Adverse effect (diplopia)	1
Adverse effect (nausea)	1
To reduce the number of oral drugs	1

### The dose of opioids before and after the discontinuation of pregabalin

Pregabalin was discontinued in 31 patients due to oral feeding difficulties. Twenty-three out of 31 patients survived 4 days or more after its discontinuation. For the 3 days before the discontinuation of pregabalin, the dose of opioid drugs was increased in three patients and unchanged in 20 patients. The dose of opioid drugs administered 24 h after the discontinuation of pregabalin was increased in seven patients, remained unchanged in six patients, and was decreased in nine patients. Furthermore, one patient discontinued opioid drugs. This patient’s medical records showed that pain was ameliorated by the effects of radiation therapy, and was controlled by an intravenous injection of NSAIDs (non-steroidal anti-inflammatory drugs).

**Fig. 1:**
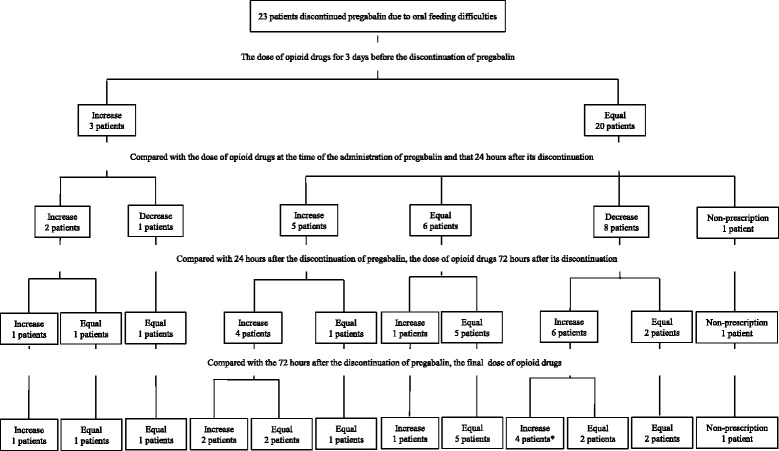
Changes in the dose of opioid drugs before and after the discontinuation of pregabalin due to oral feeding difficulties. *: Only one example had a lower dose than that before the discontinuation of pregabalin

Changes in the dose of opioid drugs administered before and after the discontinuation of pregabalin are shown in Fig. 1. The dose of opioid drugs administered was higher in 12 (52.1 %) out of 23 patients 72 h after the discontinuation of pregabalin than 24 h after. The dose of opioid drugs was increased further in eight (66.7 %) out of 12 patients 72 h after the discontinuation of pregabalin. The dose of opioid drugs administered was higher in 13 (56.5 %) out of 23 patients after pregabalin was discontinued than before (Table 5).

**Table 5:** The final dose of opioid drugs compared with that at the time of the administration of pregabalin

	Number of patients (*N*)
Increase	13
Equal	5
Decrease	4
Non-prescription (pain control by NSAIDs)	1

*NSAIDs* non-steroidal anti-inflammatory drugs

## Discussion

The results of the present study revealed that the physicians of the Department of Palliative Care mainly prescribed pregabalin to terminally ill cancer patients for pain and numbness caused by cancer (Table 2). Furthermore, the incidence of bony metastases was significantly higher in terminally ill cancer patients administered pregabalin than in those not taking the drug (Table 1), because pregabalin is effective in patients with the neuropathic pain due to bony metastases.

In the present study, the initial and maintenance doses administered were smaller than the doses described in the package insert (Table 3). Pain control was achieved without administering more than the recommended dose. The incidence of the side effects of pregabalin based on responses on an interview form was previously reported to be between 64.5 and 82.9 %. In the present study, the incidence of side effects was 18.8 % (Table 4), which was very low. Previous studies reported that the incidence of side effects was higher in the elderly and patients with renal dysfunction [5, 6].

All patients discontinued the use of oral pregabalin (Table 4), with the main reason being oral feeding difficulties. No drugs have the same effects as pregabalin, except for oral drugs. If patients have difficulties in oral feeding, the doses of opioid drugs were adjusted (Table 5, Fig. 1). The dose of opioid drugs administered 24 h after the discontinuation of pregabalin was increased in seven patients due to pain exacerbation. Furthermore, doses were gradually increased over time for some patients (Fig. 1). The dose of opioid drugs administered 24 h after the discontinuation of pregabalin was decreased in nine patients due to a deterioration in the patient status (including hepatic and renal dysfunctions) (Fig. 1). After the discontinuation of pregabalin, the dose of opioid drugs administered was increased in 56.5 % of patients with oral feeding difficulties (Table 5). For the 7 days before the discontinuation of pregabalin, the dose of pregabalin was increased in one patients and unchanged in 22 patients. Pregabalin is an effective treatment for neuropathic pain that is resistant to opioid drugs. Similar to opioid drugs, pregabalin plays an important role in the control of pain in cancer patients [1]. Isebaba et al. previously performed a questionnaire survey [7]; 52.9–75.2 % of respondents answered that the use of adjuvant analgesics was effective, while 16 % answered that the dose of opioid drugs administered may be markedly decreased in a large number of patients. Thus, we considered it important to increase the dose of opioid drugs because it is not possible to control pain in patients following the discontinuation of pregabalin.

A new dosage form to continue pain control is needed. Hospital formulations of suppositories have been investigated, and their clinical application is expected [1].

## Conclusion

Regarding the current status of pregabalin treatments in terminally ill cancer patients, all patients discontinued oral pregabalin, and the main reason was oral feeding difficulties. This study clarified that more than 50 % of patients increased the dose of opioid drugs after the discontinuation of pregabalin, with doses being repeatedly increased for some patients. A new route of administration is required for cancer patients who are unable to take oral medication.

## Abbreviations

Ccr: Creatinine clearance
NSAIDs: Non-steroidal anti-inflammatory drugs
PS: Performance status
